# Dipyridamole activates adenosine A2B receptor and AMPK/cAMP signaling and promotes myogenic differentiation of myoblastic C2C12 cells

**DOI:** 10.3389/fphar.2023.1247664

**Published:** 2023-09-12

**Authors:** Miguel Marco-Bonilla, Raquel Herencia, María Fresnadillo, Fernando Huete-Toral, Gonzalo Carracedo, Raquel Largo, Gabriel Herrero-Beaumont, Aránzazu Mediero

**Affiliations:** ^1^ Bone and Joint Research Unit, FIIS-Fundación Jiménez Díaz UAM, Madrid, Spain; ^2^ Ocupharm Group Research, Faculty of Optic and Optometry, University Complutense of Madrid, Madrid, Spain; ^3^ Department of Optometry and Vision, Faculty of Optic and Optometry, University Complutense of Madrid, Madrid, Spain

**Keywords:** adenosine, A2B receptor, muscle, sarcopenia, dipyridamole

## Abstract

**Introduction**: Sarcopenia is defined as a loss of muscle mass and strength. ATP homeostasis is crucial during myogenesis. We determined how the purinergic system modulates myogenesis using dipyridamole (blocks adenosine taken up by the cells) and tenofovir (inhibits ATP release) in a myoblast cell line.

**Methods:** C2C12 cells were differentiated in the presence/absence of tenofovir/dipyridamole, with/without the A2B selective inhibitor PSB-603. Extra-/intracellular nucleotides were examined via HPLC. The expression of muscle differentiation proteins (Pax7, Mif5, MyoD, MyoG, and MHC), PKA/CREB, adenosine receptors (A1, A2A, A2B, and A3), ATP-channel pannexin-1 and the P2X7 receptor was analyzed via WB and RT-PCR. cAMP and AMPK activation was measured.

**Results:** Tenofovir increased intracellular ATP and reduced extracellular adenosine, decreasing Pax7 expression and increasing MHC expression prematurely. Dipyridamole increased intracellular AMP and extracellular adenosine, counteracting the premature myogenesis promoted by tenofovir. All adenosine receptors were expressed during differentiation with dipyridamole, increasing A2B expression. Tenofovir maintained inactive AMPK and decreased cAMP levels, as well as PKAα and pCREB expression, which were recovered with dipyridamole.

**Discussion:** Adenosine and ATP act as mediators in muscle myogenesis. The blockade of ATP release by tenofovir promotes premature myogenesis, with dipyridamole counteracting the premature differentiation promoted by tenofovir via the adenosine A2B receptor and cAMP/AMPK pathways. Therefore, dipyridamole might be of interest as a therapeutic approach in sarcopenia.

## Graphical Abstract

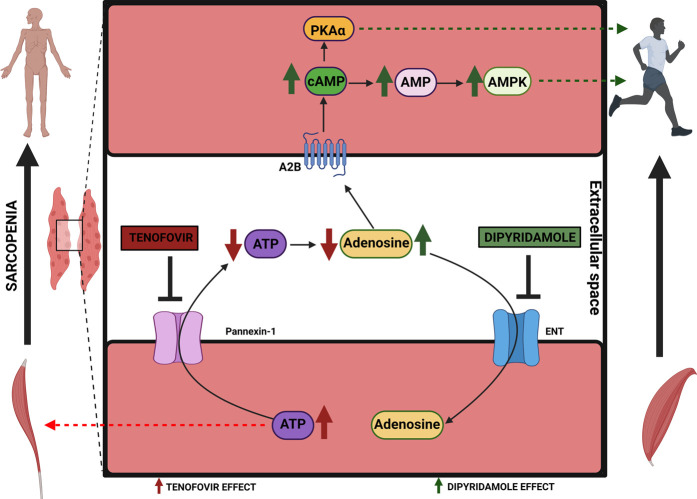

## 1 Introduction

Sarcopenia is defined as a generalized and progressive loss of skeletal muscle mass and muscle function (Santilli et al., 2014). It can be either primary, associated with aging, or secondary, when causal factors are identified, such as a chronic inflammatory disease and/or organ failure (Dhillon and Hasni, 2017). Muscle loss in primary and secondary sarcopenia appears to be driven by different mechanisms: sarcopenia in the elderly is primarily produced by anabolic resistance induced by myostatin (Bergen et al., 2015); meanwhile, secondary sarcopenia appears to be driven by catabolic processes (Sharma and Dabur, 2020). During inflammation, there is a shift in skeletal muscle homeostasis toward muscle loss.

5′-Adenosine monophosphate-activated protein kinase (AMPK) plays an important role in the control of skeletal muscle development and growth (Jeon, 2016; Herzig and Shaw, 2018). AMPK activation is mediated by phosphorylation at Tyr172 of the α-subunit (Willows et al., 2017). AMPK is a serine/threonine protein kinase that regulates cellular energy homeostasis, acting as a central energy sensor that maintains energy stores by fine-tuning anabolic and catabolic pathways. AMPK activation rewires metabolism to decrease anabolic processes (ATP consumption) and increase catabolism (ATP production) to restore a more favorable energy balance (Willows et al., 2017). AMPK activation is generally suppressed in sarcopenic muscles (Wright et al., 2019).

The purinergic system is a signaling system, where purine nucleotides and the nucleoside adenosine act as messengers (Burnstock, 1997). Adenosine activates the G protein-coupled receptors A1, A2A, A2B, and A3 (10, 11). The A1 and A3 receptors inhibit the production of cyclic AMP (cAMP) through Gi, and the A2A and A2B subtypes are coupled to Gs/Go to stimulate adenylate cyclase and cAMP (Sachdeva and Gupta, 2013; Vecchio et al., 2017). Intracellular ATP is released from the cell by pannexin-1 (Panx-1) channels. The released ATP reaches the P2 ionotropic (P2X) and metabotropic (P2Y) receptors (Burnstock, 2007). The distribution and expression of adenosine and ATP receptors are essential in muscle. The absence of the adenosine A1 receptor was observed in humans (Zheng et al., 2007). The presence of the adenosine A2A and A2B receptors was detected in the cytosol and membrane of human skeletal muscle cells (Zheng et al., 2007). The A2B receptor is abundantly expressed in muscle compared to the rest of the adenosine receptors (Gnad et al., 2020). In addition, A2B knockout leads to a decrease in satellite cells, producing a sarcopenic-like phenotype (Gnad et al., 2020). The adenosine A3 receptor has a dual role (proangiogenic/proapoptotic) dependent on the tissue type and adenosine concentration. Concentrations of <25 nM favor apoptosis, while concentrations of >100 nM increase proliferation in *in vitro* models of melanoma and leukemia (Mazziotta et al., 2022). On the other hand, Panx-1 has been implicated in muscle fusion and migration (Langlois et al., 2014). The P2X7 receptor has been detected in myoblasts and myotubes and is related to myotube branching, expansion and satellite cell proliferation/differentiation, preventing skeletal muscle atrophy (Banachewicz et al., 2005; Fabbrizio et al., 2020). The expression of several P2X and P2Y receptors have been demonstrated in developing rat, chicken and mouse skeletal muscle and C2C12 cells (Burnstock, 2007). Moreover, P2Y4 and P2Y11 receptors were expressed in the sarcolemma and intracellularly, respectively, in human skeletal muscle fibers (Bornø et al., 2012). Additionally, P2Y6 was found in the mouse C2C12 myoblast cell line (Balasubramanian et al., 2014; Nishimura et al., 2017).

There are commercially available pharmacological compounds that modulate the purinergic system. Tenofovir (an analogue of AMP and a potent inhibitor of the HIV reverse transcriptase) has been shown to exert inhibitory activity on Panx-1, compromising the release of ATP into the extracellular space (Feig et al., 2017; Conesa-Buendía et al., 2019). Dipyridamole (an antiplatelet that inhibits PDE3) is able to inhibit nucleoside transport (ENT1, ENT2, and ENT4), producing an increase in extracellular adenosine levels through inhibition of adenosine taken up by the cells (Ward et al., 2000; Ramakers et al., 2011). Recently, we observed that dipyridamole reverted the bone deleterious effect of tenofovir both *in vitro* and *in vivo* (Conesa-Buendía et al., 2019). We observed that mice treated with tenofovir lost nearly 10% of their body weight, which was recovered when the mice were treated with dipyridamole (Conesa-Buendía et al., 2019).

Considering these findings, in this study, we aimed to determine the involvement of the purinergic system in muscle maintenance and thereby elucidate the intracellular pathways involved. For this purpose, we used tenofovir and dipyridamole as compounds to block ATP transport and adenosine uptake to modulate the intra/extracellular nucleotide concentrations to determine if those changes have any effect in myogenesis.

## 2 Materials and methods

### 2.1 Cell culture

C2C12 cells (#CRL-1772, American Type Culture Collection, ATCC, Manassas, VA, United States) were cultured in DMEM (Corning, Cultek, Spain, #10–013) supplemented with 10% FBS (Gibco, #11573397) and 1% penicillin–streptomycin (Sigma, #P4333). To induce myogenic differentiation, 70% confluence C2C12 cells were cultured in DMEM containing 2% HS (Gibco, #11540636) (Wong et al., 2020). Cells were maintained in a humidified chamber at 37°C, in 95% air and 5% CO_2_. Media were replaced with fresh media every 48 h.

### 2.2 Cell proliferation assay

C2C12 cells were plated in 96-well plates (Corning, Cultek, Spain) (12.500 cells/well). Cell proliferation was measured using an AlamarBlue (Bio-Rad, #BUF012B) assay according to the manufacturer’s instructions, after 24 and 48 h of myogenic differentiation (Czekanska, 2011). Cells were treated with 10^−9^–10^−5^ M tenofovir (Sequoia Research Products (Carbosynth Limited, Berkshire, United Kingdom, #FT10480) or dipyridamole (Sigma-Aldrich,Madrid, Spain, #D9766).

### 2.3 RNA isolation and quantitative real-time polymerase chain reaction (RT-PCR)

C2C12 cells were treated with tenofovir, dipyridamoleor a combination of both (1 μM each) in 2% HS for 4 days. Total RNA was extracted using TRIzol reagent and retrotranscribed using a MuLV Reverse transcriptase PCR kit (Applied Biosystems, Foster City, CA, United States). Relative quantification of gene expression was performed via real-time RT-PCR on a Step One Plus (Applied Biosystems) with Power UP SYBR Green MasterMix according to the manufacturer’s protocol. The primers listed in Table 1 were used. The Pfaffl method was used for relative quantification (Pfaffl, 2001).

**TABLE 1:** Primers used in the RT-PCR reaction.

Gene	Forward primer	Reverse primer
*A1*	TGT​GCC​CGG​AAA​TGT​ACT​GG	TCT​GTG​GCC​CAA​TGT​TGA​TAA​G
*A2A*	AGC​CAG​GGG​TTA​CAT​CTG​TG	TAC​AGA​CAG​CCT​CGA​CAT​GTG
*A2B*	AGC​TAG​AGA​CGC​AAG​ACG​C	GTG​GGG​GTC​TGT​AAT​GCA​CT
*A3*	AAG​GTG​AAA​TCA​GGT​GTT​GAG​C	AGG​CAA​TAA​TGT​TGC​ACG​AGT
*PANX-1*	GTG​GAG​AAG​AGG​GTC​TGT​GC	GAA​AAC​CCC​AGC​CAA​GAG​GA
*P2X7*	GAC​AAA​CAA​AGT​CAC​CCG​GAT	CGC​TCA​CCA​AAG​CAA​AGC​TAA
*18S*	GGGAGCCTGAGAAACGGC	GGG​TCG​GGA​GTG​GGT​AAT​TT

### 2.4 Western blot

C2C12 cells were treated with tenofovir, dipyridamoleor a combination of both (1 μM each) with/without PSB-603 1 µM (Tocris, #3198) in 2% HS for up to 4 days (Llamas-Granda et al., 2021). Also C2C12 were treated with adenosine 7.5 µM (Sigma Aldrich, #4036), EHNA 1 µM (Cayman Chemicals, #13352) or ATP 100 µM (Sigma Aldrich, #2383) for 24 h and 4 days of differentiation. Cells were lysed with RIPA buffer, and the protein concentration was determined using BCA. An amount of 4–10 µg of protein was subjected to 6%–15% SDS-PAGE and transferred to a nitrocellulose membrane. Membranes were blocked with 3% BSA in Tris-buffered saline containing 0.1% Tween-20 (TBS-T). Membranes were incubated overnight at 4°C with the primary antibodies for adenosine A1 (#55026-1-AP), Panx-1 (#12595-1-AP), CD39/ENTPD1 (#19229) (1:500), NT5E/CD73 (#12231) (1:1,000) (Proteintech, Rosemont, United States), A2A (# BS-1456R), A2B (#PA5-72850), A3 (#PA5-33326), MHC (#MA5-32555), PKAγ (#A25933) (Thermo Fisher Scientific, Waltham, United States), P2X7 (#ab109054), PKAβ (#ab187515), Mif5 (1:200) (#ab125078), MyoD (1:200) (#ab125078), myogenin (1:200) (#ab124800) (Abcam, Cambridge, United Kingdom), AMPK (#2532), pAMPK (Thr172) (#2535), PKAα (#4782), pCREB (Ser133) (#ab32096), CREB (#ab32515) (Cell signaling, Danvers, EEUU) (1:1,000 each) and Pax-7 (#AB_528428) (Developmental Studies Hybridoma Bank, Houston, EEUU) (1:500). After washed in TBS-T and incubated with the secondary antibody anti-rabbit IgG HRP (Cytiva, #NA934V) or anti-mouse IgG HRP (Cytiva, #NA931V) (1:1,000 each) for 1 h, immune complexes were visualized using immobilon HRP substrate (Millipore, Danvers, United States) and acquired using an Amersham Imager 600 (GE Healthcare Life Sciences). Reprobing with anti-actin mouse (Santa-Cruz, #sc-47778), anti-actin rabbit (Sigma Aldrich, #A5060) and tubulin (Sigma Aldrich, #T5168) was performed to check that all lanes were loaded with the same amount of protein. Digital densitometric band analysis was performed using the Quantity One software (Bio-Rad, Madrid, Spain), and band intensities were expressed relative to actin. Variations in intensity are expressed as % of basal (day 0) and as the mean ± SEM. All results were calculated as a percentage of the non-differentiated controls to minimize the intrinsic variation among the different experiments.

### 2.5 cAMP concentration

C2C12 cells were seeded at 200.000 cells/well, and on days 0 and 4 of differentiation, the cells were treated with 1 μM each of tenofovir and dipyridamole for up to 2 h (Suh et al., 2000). The cAMP concentration was measured using a colorimetric cAMP ELISA kit following the manufacture’s protocol for acetylated cell lysates (Cell Biolabs, San Diego, United States). Absorbance was measured at 450 nm using a microplate reader, Tecan Spark 20 M.

### 2.6 AMPK activity

C2C12 cells were seeded at 12.500 cells/well in a 96-well black (clear-bottom) plate, and on days 0 and 4 of differentiation, the cells were treated with 1 μM each of tenofovir and dipyridamole for up to 3 h. The assay was performed with an AMPK phosphorylation assay kit (Abnova Corporation, Jhouzih St, Taiwan) according to the manufacturer’sinstructions, and pAMPK was measured at λex/em 530/585 nm and 360/450 nm for total protein with a Tecan Infinite.

### 2.7 Determination of nucleotide concentration via HPLC

C2C12 cells were treated with tenofovir, dipyridamole or a combination of both (1 μM each) in 2% HS for up to 4 days. The supernatants and cell lysates were collected every day. The cells were lysed with a homemade buffer (1 mM phenylmethylsulfonyl fluoride, 1 mM protease inhibitor cocktail (#p8340, Sigma Aldrich), 1 mM sodium orthovanadate, 1 mM sodium fluoride and 1 mM β -glicerophospate), and the supernatants were used directly. Samples were treated with EHNA and dipyridamole 1 µM each to avoid adenosine degradation/cellular uptaken. Protein denaturation and HPLC analysis were performed as described previously by Vivero-Lopez et al. (Vivero-Lopez et al., 2022). Briefly, a heat shock step was carried out at 98°C for 2 min. All samples were centrifuged at 13000 g for 10 min at 4°C, and the supernatants were collected and stored at −80°C until use. Inosine, adenosine, AMP, ADP, and ATP concentrations were determined via high-performance liquid chromatography (HPLC) using a liquid chromatography with a reversed phase column (Agilent 1,100 Series Liquid Chromatography) and a UV detector set at 254 nm. The buffer composed of 0.1 mol/L KH_2_PO_4_ (pH 7.5) and 18% acetonitrile was run at 1.5 mL/min for 20 min. Compounds were identified and quantified by their retention times and peak areas of known standards, calibrated via spectrophotometry. The results are expressed as the mean ± SEM. All results were corrected according to heat shock lost and calculated as a percentage of the controls without a differentiation state.

### 2.8 Statistical analysis

The statistical significance of the differences among groups was determined with the use of one-way ANOVA and Bonferroni’spost hoctest. All statistics were calculated using GraphPad software (La Jolla, CA, United States).

## 3 Results

### 3.1 Tenofovir alters the myoblastic proliferative state

Previously, we demonstrated that 1 µM dipyridamole reverted the bone deleterious effects of 1 µM tenofovir (Conesa-Buendía et al., 2019). To corroborate whether the same doses could be used in the present study, we conducted a dose–response proliferation assay. Twenty-4 h after the addition of 2% horse serum (HS), tenofovir showed a dose–response inhibition during proliferation compared to the control (63.3 ± 5% 1 µM tenofovir and 62.8 ± 6.4% 10 µM tenofovir vs. 100 ± 7.3% control, *p < 0.05*), which was increased after 48 h (Supplementary Figure S1). No significant changes were observed at any dose in the presence of dipyridamole after both 24 and 48 h of incubation (Supplementary Figure S1). Therefore, we used 1 µM for both compounds for all experiments.

### 3.2 Tenofovir and dipyridamole sufficiently modulate adenosine nucleotide concentrations as well as muscle differentiation markers

**FIGURE 1:**
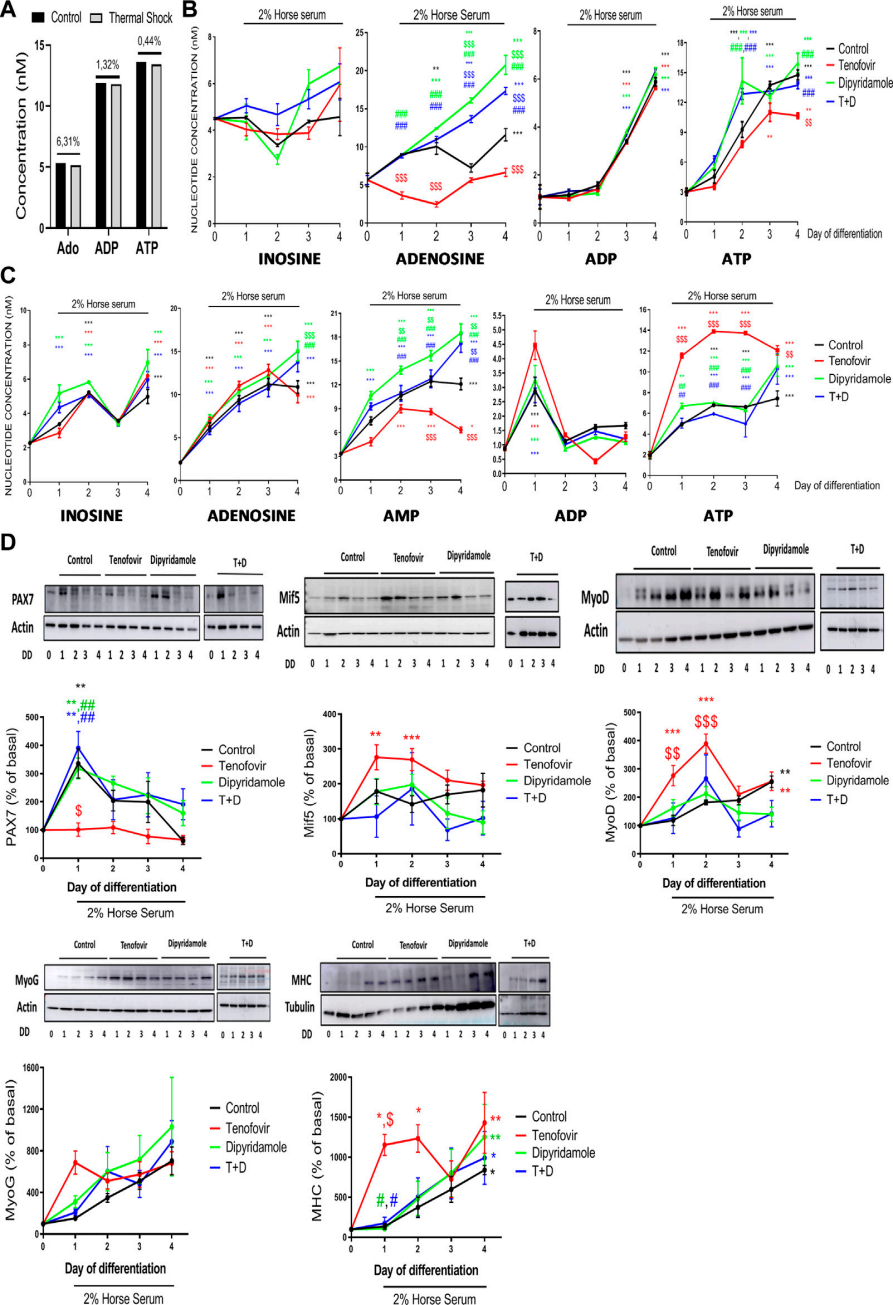
Analysis via high-performance liquid chromatography (HPLC) over 4 days of differentiation in the presence of tenofovir and dipyridamole. **(A)** Loss of nucleotides due to the thermal shock process. **(B)** Extracellular concentrations of inosine, adenosine, AMP, ADP and ATP. **(C)** Intracellular concentrations of inosine, adenosine, AMP, ADP and ATP assessed via HPLC. **(D)** Western blot analysis of myoblast/myotube markers. One representative of *n* = 5 per group and day is displayed. Data are the mean ± SEM.*: treatment vs. basal; $: tenofovir and dipyridamole vs. control; #: dipyridamole and T + D vs. tenofovir. T + D: tenofovir + dipyridamole; DD: days of differentiation.

To understand whether the dipyridamole and tenofovir treatments induced changes in adenosine nucleotide concentrations that were sufficient to modulate myogenesis, we analyzed them via HPLC. The heat shock process induced a loss of adenosine, ADP and ATP (6.31%, 1.32% and 0.44% losses for adenosine, ADP and ATP standards that were not heat-shock-processed vs. heat-shock-processed standards, respectively) that was taken into consideration (Figure 1A), and a correction value corresponding to the heat shock loss was applied in the analysis.

At the extracellular level, inosine is modulated within time of myotube differentiation, but no significant changes were observed when compare to myoblast, and its values were not modified with any treatment (Figure 1B). Extracellular adenosine increased during differentiation (11.6 ± 0.81 nM control on day 4 vs. 5.65 ± 0.88 nM basal, *p < 0.001*), being significant on day 2 (*p < 0.005*) (Figure 1B). Tenofovir decreased extracellular adenosine concentrations during differentiation (6.65 ± 0.54 nM tenofovir vs. 11.6 ± 0.81 nM control on day 4, *p < 0.001*), being significant on days 1 and 2. Dipyridamole treatment increased the levels of extracellular adenosine (*p < 0.001*), being significant compared to the control on days 3 and 4 (17.33 ± 0.47 nM dipyridamole vs. 11.6 ± 0.81 nM control on day 4, *p < 0.001*) (Figure 1B), and compared to tenofovir from days 1–4 (*p < 0.001*), as the combined treatment increased extracellular adenosine levels, similar to dipyridamole alone (Figure 1B). No measurable levels of extracellular AMP were detected in the supernatant. In the presence of 2% HS, the ADP levels remained constant until day 2 and increased from day 3 (*p < 0.001*) of differentiation (5.88 ± 0.18 nM control on day 4 vs. 1.09 ± 0.49 nM basal, *p < 0.001*, Figure 1B), and they were not modulated by the treatments (*p = ns*). Extracellular ATP levels increased during differentiation (14.7 ± 0.5 nM control on day 4 vs. 3.02 ± 0.3 nM basal, *p < 0.001*), being significant on days 2 (*p < 0.001*) and 3 (*p < 0.001*) (Figure 1B). Tenofovir reduced extracellular ATP levels on day 4 (10.67 ± 0.28 nM tenofovir on day 4 vs. 14.7 ± 0.5 nM control on day 4, *p = 0.005*, Figure 1B). Dipyridamole did not modify the extracellular ATP levels compared to the control, but it reverted the effect of tenofovir when both were combined (13.21 ± 0.22 nM T + D vs. 10.67 ± 0.28 nM tenofovir on day 4, *p < 0.001*, Figure 1B).

When we analyzed the intracellular nucleotide levels, we observed that 2% HS increased the inosine levels (4.98 ± 0.41 nM control on day 4 vs. 2.27 ± 0.04 nM basal, *p < 0.001*), being significant on day 2 (*p < 0.001*) (Figure 1C). Tenofovir did not modulate these levels (*p = ns*); meanwhile, dipyridamole increased the inosine levels on day 4 (14.17 ± 1.75 nM dipyridamole vs. 11.07 ± 0.54 nM control, *p < 0.001*, Figure 1C), but it was not able to revert the effect of tenofovir (*p = ns*). Intracellular adenosine increased progressively over the 4 days of differentiation (10.87 ± 0.73 nM control on day 4 vs. 2.10 ± 0.19 nM basal, *p < 0.001*, Figure 1C). Tenofovir did not change the intracellular adenosine levels compared to the control (*p = ns*), but dipyridamole increased them on day 4 of differentiation (13.76 ± 1.13 nM dipyridamole vs. 10.87 ± 0.73 nM control on day 4, *p < 0.001*) and reverted the effect of tenofovir (*p = 0.001*, Figure 1C). The intracellular AMP levels increased during differentiation (12.10 ± 0.73 nM control on day 4 vs. 3.33 ± 0.18 nM basal, *p < 0.001*), being significant from day 1 (*p < 0.001*) (Figure 1C). Tenofovir reduced the AMP levels compared to the control, being significant on days 3 and 4 (6.33 ± 0.35 nM tenofovir vs. 12.1 ± 0.73 nM control on day 4, *p < 0.001*, Figure 1C). Dipyridamole increased intracellular AMP (18.53 ± 1.13 nM dipyridamole vs. 12.10 ± 0.73 nM control on day 4, *p < 0.001*), being significant on days 1–3 (*p < 0.001*), and it reverted the effect of tenofovir (*p < 0.001*) (Figure 1C). The ADP levels increased on day 1 in the presence of 2% HS (2.86 ± 0.49 nM control vs. 0.87 ± 0.04 nM basal, *p < 0.001*), and the treatments did not modulate these levels (Figure 1C). The intracellular ATP levels increased with 2% HS on day 2 (7.44 ± 0.73 nM control vs. 1.94 ± 0.35 nM basal, *p < 0.001*) and remained constant until day 4 (*p < 0.001,* Figure 1C). Tenofovir increased intracellular ATP starting from day 1 of differentiation compared to the control (11.56 ± 0.26 nM tenofovir vs. 4.93 ± 0.02 nM control, *p < 0.001*, Figure 1C). Dipyridamole maintained similar intracellular ATP levels to the control and reverted the effect of tenofovir (4.13 ± 0.44 nM T + D vs. 11.74 ± 0.08 nM tenofovir on day 1, *p < 0.005*, Figure 1C).

Next, myogenesis markers were studied to analyze whether the observed changes in intra/extracellular nucleotides were able to modulate them. As expected, PAX7 increased on the first day of induction with 2% HS (315% ± 58% control on day 1 vs. basal, *p < 0.05*, Figure 1D), being reduced to basal levels during differentiation. Tenofovir decreased PAX7 expression to basal levels (101% ± 23% tenofovir vs. 315% ± 58% control on day 1, *p < 0.05*, Figure 1D), and dipyridamole was able to revert the effect to control levels (*p < 0.005*, Figure 1D). The Mif5 levels remained stable during differentiation (*p = ns*), with tenofovir increasing them during the first 2 days of differentiation (142% ± 23% tenofovir vs. basal, *p* < 0.001, Figure 1D). Dipyridamole did not modulate Mif5 expression (*p = ns*). MyoD protein expression showed a progressive increase during differentiation (255% ± 20% control on day 4 vs. basal, *p < 0.005*, Figure 1D) that was enhanced by tenofovir (389% ± 33% tenofovir vs. 182% ± 8% control on day 2, *p < 0.001*, Figure 1D) and reverted by dipyridamole (162% ± 29% dipyridamole vs. 389% ± 33% tenofovir, *p < 0.001*, Figure 1D). MyoG expression increased with 2% HS (*p = ns*) but was not modulated by the treatments (*p* = ns). MHC was expressed at the end of cell differentiation as expected (839% ± 57% control on day 4 vs. basal, *p < 0.05*, Figure 1D). Tenofovir led to a premature expression of MHC (1,429% ± 379% tenofovir vs. 839% ± 57% control on day 1, *p < 0.05*, Figure 1D), with dipyridamole being able to revert the effect (175% ± 76% T + D vs. 1,153% ± 131% tenofovir, *p < 0.05*, Figure 1D).

**FIGURE 2:**
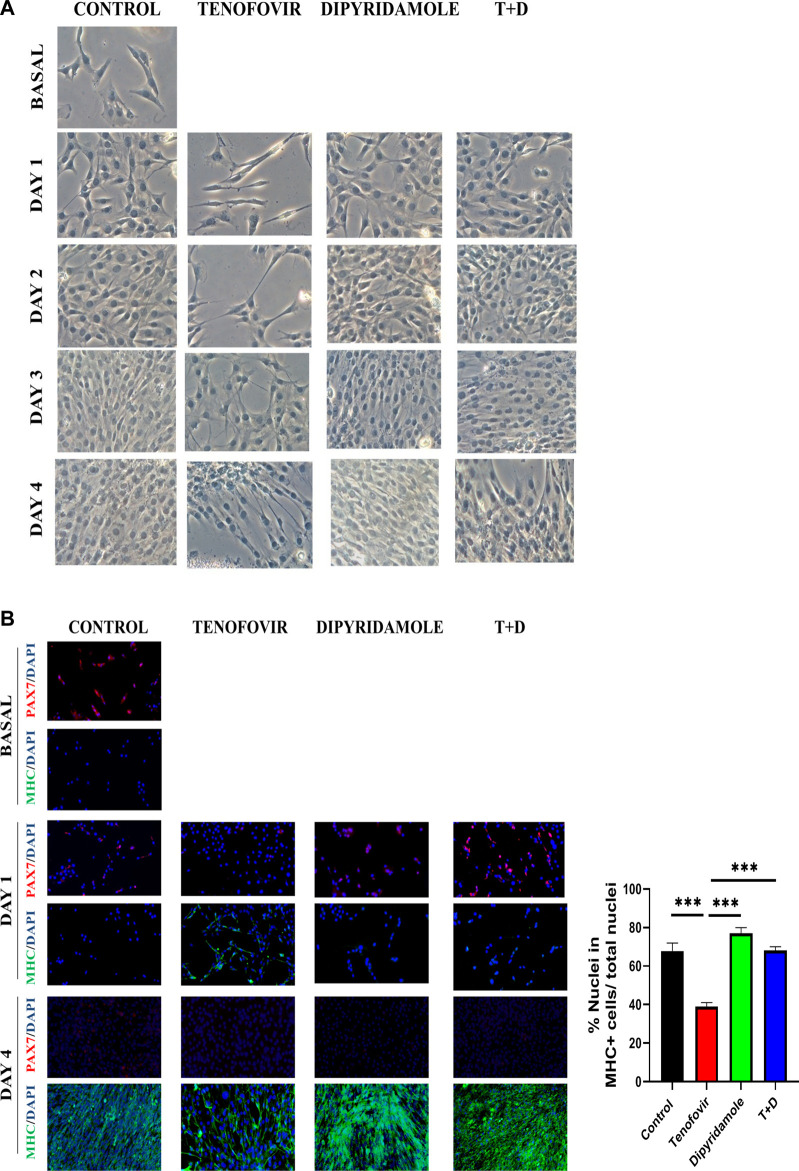
Bright field and inmunofluorescence of C2C12 myogenesis. **(A)** Bright field of C2C12 during 4 days of differentiation by 2% HS. **(B)** Inmunofluorescence of Pax7/MHC markers in C2C12 at basal and days 1 and 4 of differentiation with treatments. We show a representative picture of *n* = 5 (mean ± SEM). *: differences between treatments.

To corroborate the premature expression in MHC in the presence of tenofovir and therefore myotube formation, we analyzed morphology of muscle cells. Bright field and immunofluorescence studies showed that C2C12 cells started to form mature myotubes at 3 and 4 days of differentiation in the presence of 2% HS (Figure 2). Treatment with tenofovir led to the formation of myotubes with positive staining for MHC on day 1 of differentiation, and by day 4, these myotubes exhibited a thinner and less fused appearance compared to the control (Figure 2). Co-treatment with dipyridamole reversed this effect, showing a myogenesis pattern similar to that observed in control (Figure 2).

### 3.3 Purinergic receptors are modulated during myoblast differentiation

**FIGURE 3:**
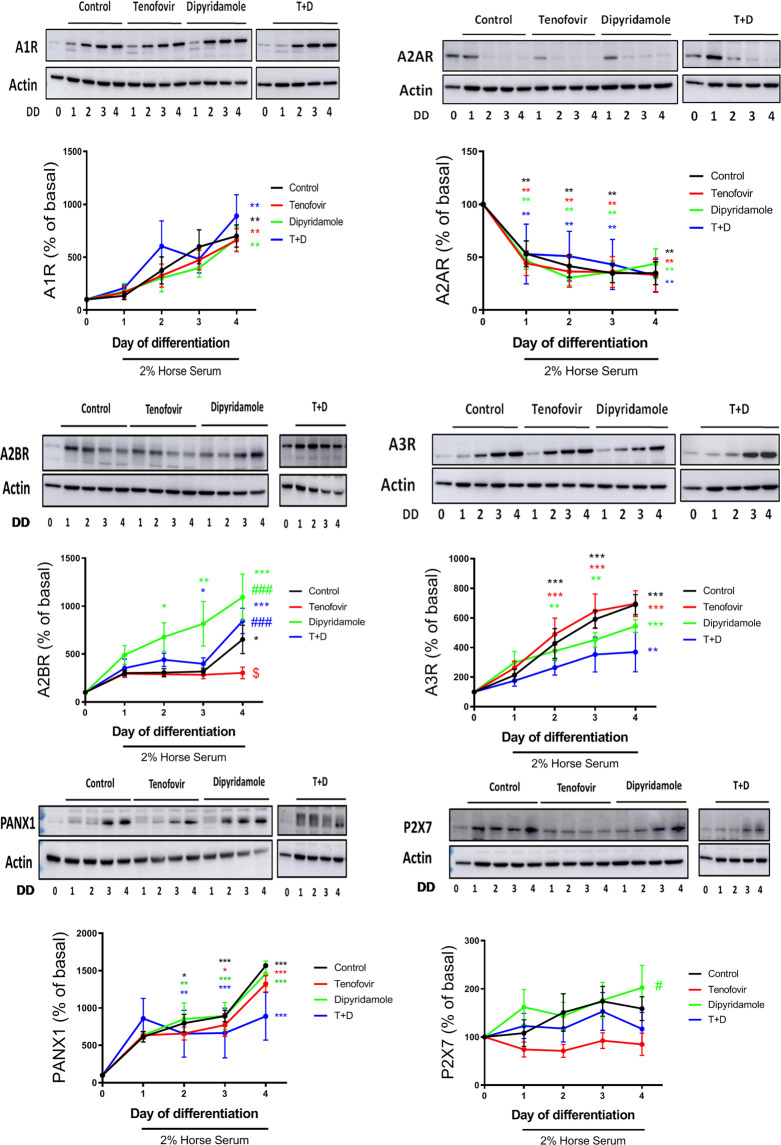
Western blot protein expression over the 4 days of differentiation in the presence of tenofovir and dipyridamole. Protein expression of the A1, A2A, A2B, and A3 adenosine receptors, P2X7 receptor and Panx-1 is shown. Data are representative of *n* = 5 each group per day (mean ± SEM). *: treatment vs. basal; $: tenofovir and dipyridamole vs. control; #: dipyridamole and T + D vs. tenofovir. T + D: tenofovir + dipyridamole; DD: days of differentiation.

The expression of adenosine receptors, the P2X7 receptor and the Panx-1 channel was analyzed during myoblast differentiation. A1 receptor protein expression increased significantly during differentiation (699% ± 105% control vs. basal, *p < 0.005*, Figure 3), with no modulation by the treatments (*p = ns*). Adenosine A2A receptor protein expression decreased during differentiation (34.9% ± 10.8% control on day 4 vs. basal, *p < 0.005*, Figure 3), being significant from day 1 (*p < 0.005*), and the treatments did not modulate this protein expression (*p = ns*). Adenosine A2B receptor protein expression increased with 2% HS (652% ± 148% control on day 4 vs. basal, *p < 0.05*, Figure 3). The treatment with tenofovir reduced the expression of adenosine A2B receptor at day 4 of differentiation (303% ± 60% tenofovir vs. 553% ± 47% control, *p < 0.05*, Figure 3) and dipyridamole increased A2B protein expression and prevented the effect of tenofovir (884% ± 132% T + D vs. 553% ± 47% control and 303% ± 60% tenofovir on day 4, *p < 0.05* and *p < 0.001*, respectively, Figure 3). A3 receptor protein expression increased with 2% HS (689% ± 68% control on day 4 vs. basal, *p < 0.001*, Figure 3), being significant on days 2 and 3 (*p < 0.001*). Tenofovir did not change A3 expression when compared to the control as well as dipyridamole alone or in combination (*p = ns*) (Figure 3). Furthermore, 2% HS increased Panx-1 protein expression (1,567% ± 30% control on day 4 vs. basal, *p < 0.001*, Figure 3), but this protein expression was not changed by the treatments (*p = ns*). Finally, 2% HS did not change P2X7 protein expression during differentiation (*p = ns*), and tenofovir did not module P2X7 expression (*p = ns*, Figure 3). However, dipyridamole increased P2X7 expression compared to tenofovir (202% ± 46% dipyridamole vs. 89% ± 19% tenofovir on day 4, *p < 0.05*, Figure 3). Similar changes were observed at the mRNA levels (Supplementary Figure S2).

**FIGURE 4:**
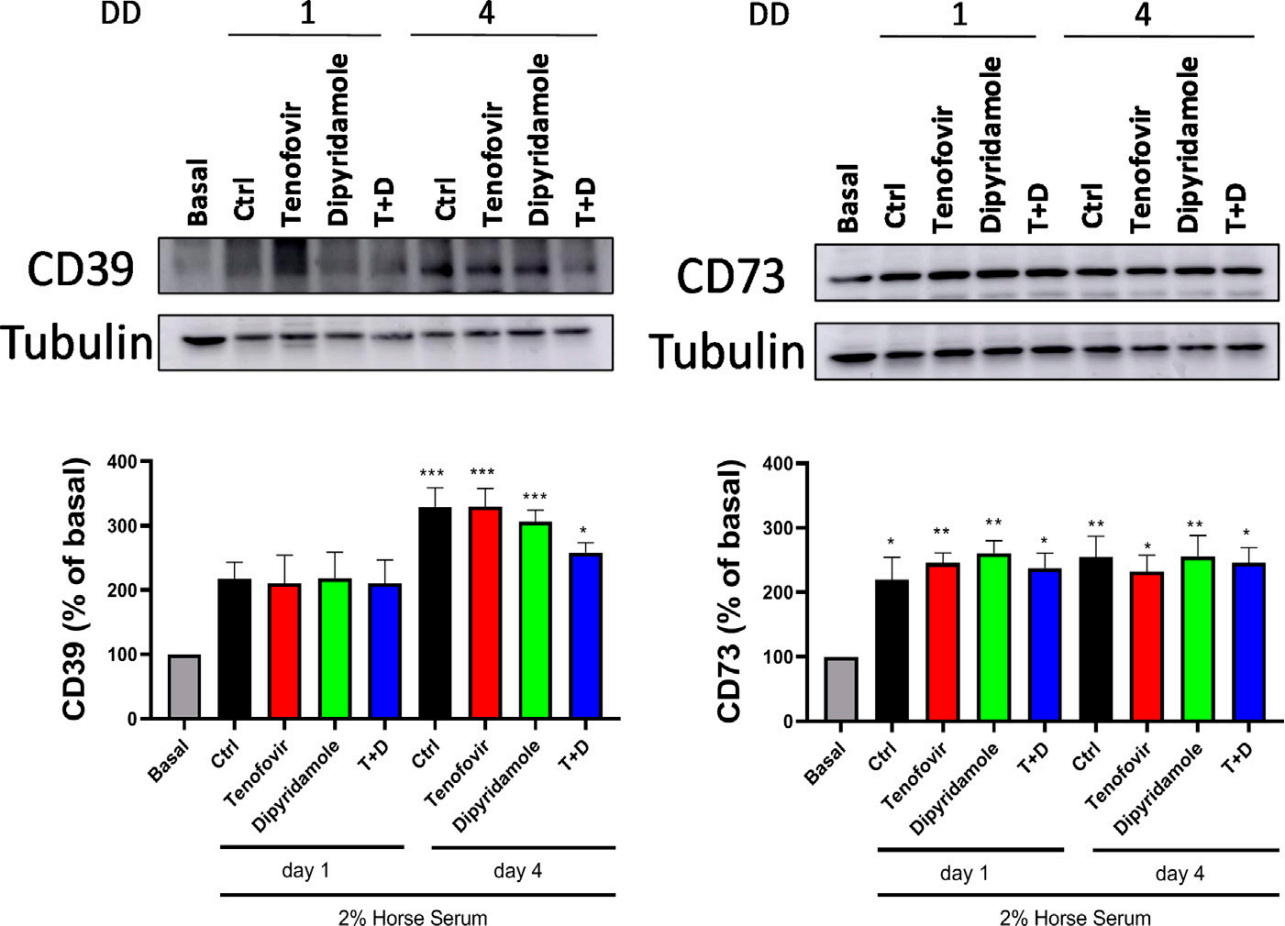
Western blot protein expression over 1 and 4 days of differentiation in the presence of tenofovir and dipyridamole. Protein expression of the CD39 and CD73 is shown. Data are representative of *n* = 5 each group per day (mean ± SEM). *: treatment vs. basal. T + D: tenofovir + dipyridamole; DD: days of differentiation.

Next, we evaluated the differential protein expression of ectonucleotidases. When CD39 protein expression was analyzed, we observed an increased expression of this enzyme within myoblast differentiation in the presence of 2% HS (217.6% ± 25.56% control day 1% and 329.1% ± 30.15% control day 4 vs. basal, *p = ns and p < 0.0005 respectively)*, with no modulation by any treatments (*p = ns*) (Figure 4). Similar trend was observed for CD73 protein expression, been increased in the presence of 2% HS (219.4% ± 35.11% control day 1% and 254.6% ± 32.53% control day 4 vs. basal, *p < 0.05 and p < 0.005 respectively*), with no difference among treatments (*p = ns*) (Figure 4).

### 3.4 Dipyridamole increases cAMP and promotes the PKAα activation

**FIGURE 5:**
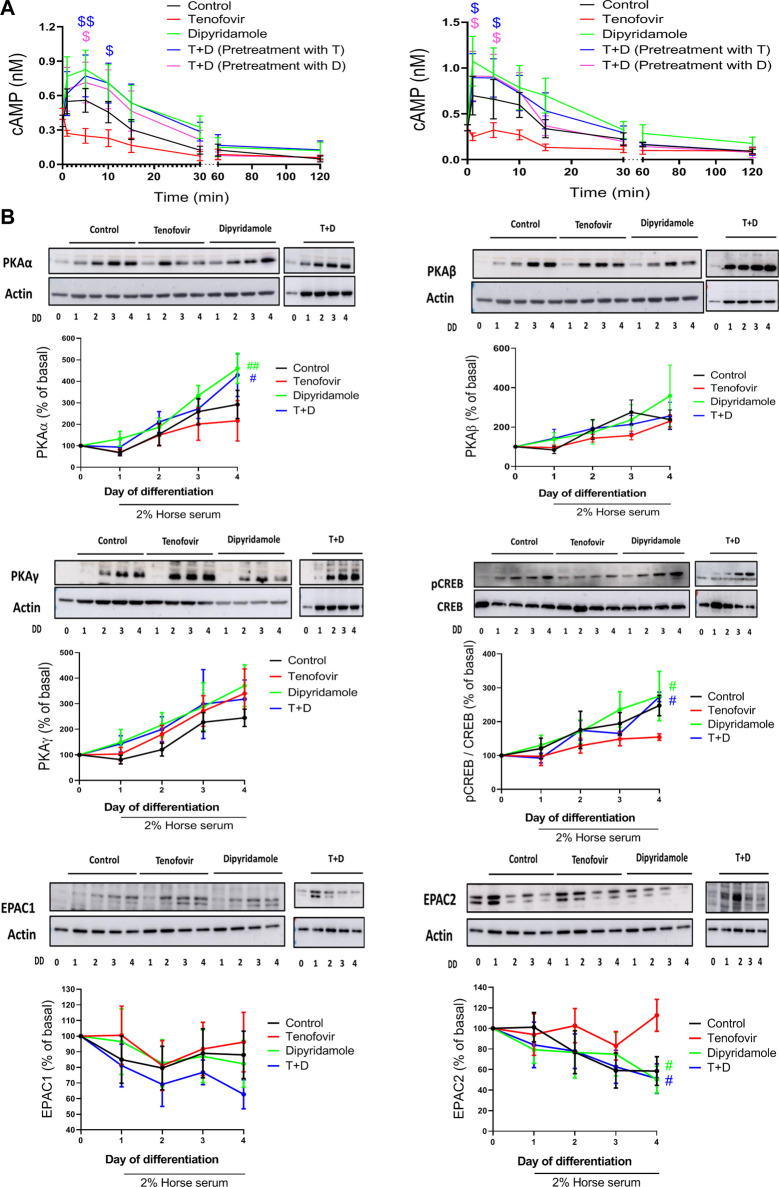
cAMP/PKA pathway and AMPK activation in the presence of dipyridamole.**(A)** cAMP levels in myoblasts (left) and myotubes (right). **(B)** Study of protein expression of PKAα, PKAβ, PKAγ, pCREB and EPAC 1 and 2. Data are representative of *n* = 5 each group per day (mean ± SEM).*: treatment group vs. control group; #: treatment group vs. tenofovir group. T + D: tenofovir + dipyridamole; DD: days of differentiation.

The cAMP signaling cascade has been linked to a delay in the onset of age-related muscle loss. When we analyzed cAMP activation at early time points, we observed an increase in the cAMP levels 5 min after 2% HS incubation (0.58 ± 0.13 nM control vs. basal, *p = 0.005*, Figure 5A) that progressively decreased with time. Dipyridamole increased cAMP after 5 min, which was maintained up to 15 min (0.85 ± 0.17 nM dipyridamole vs. 0.58 ± 0.13 nM control after 5 min, *p = 0.005*, Figure 5A), and tenofovir reduced the cAMP levels (0.24 ± 0.07 nM tenofovir vs. 0.58 ± 0.13 nM control after 5 min, *p = 0.005*, Figure 5A). The combined treatment (both dipyridamole and tenofovir pre-treatments) showed similar cAMP values to dipyridamole alone (Figure 5A). Similar results were obtained when C2C12 cells were differentiated to myotubes for 4 days and then treated with tenofovir and dipyridamole (Figure 5A).

To study the cAMP signaling pathway, we analyzed the expression of PKA on its three catalytic isoforms PKAα, PKAβ and PKAγ as well as EPAC1/2. PKAα increased during differentiation (292 ± 65% control on day 4 vs. basal, *p = 0.05*, Figure 5B), and tenofovir reduced this expression (215 ± 90% tenofovir vs. basal, *p = ns*, Figure 5B), with dipyridamole being able to revert this effect (429 ± 99.4% T + D vs. 215.8 ± 94.2% tenofovir on day 4 of differentiation, *p < 0.05*) in a similar manner to dipyridamole alone (Figure 5B). PKAβ and PKAγ expression increased during differentiation, but no changes were observed with any treatment (Figure 5B). pCREB increased during differentiation (247 ± 30% control on day 4 vs. basal, *p = 0.05*, Figure 5B). Tenofovir reduced its phosphorylation (154 ± 9% tenofovir vs. 247 ± 30% control on day 4, *p < 0.05*, Figure 5B). Dipyridamole increased pCREB, similar to the control (275 ± 73% of dipyridamole on day 4 vs. basal, *p = 0.05*, Figure 5B), and reverted the effect of tenofovir (*p < 0.05*, Figure 5B). We observed no changes in EPAC1 but a decrease in EPAC2 during differentiation, with no differences among the treatments (Figure 5B); meanwhile, EPAC2 was decreased with 2% HS and dipyridamole, and tenofovir maintained the expression similar to basal levels (Figure 5B).

### 3.5 Dipyridamole improves AMPK activation

**FIGURE 6:**
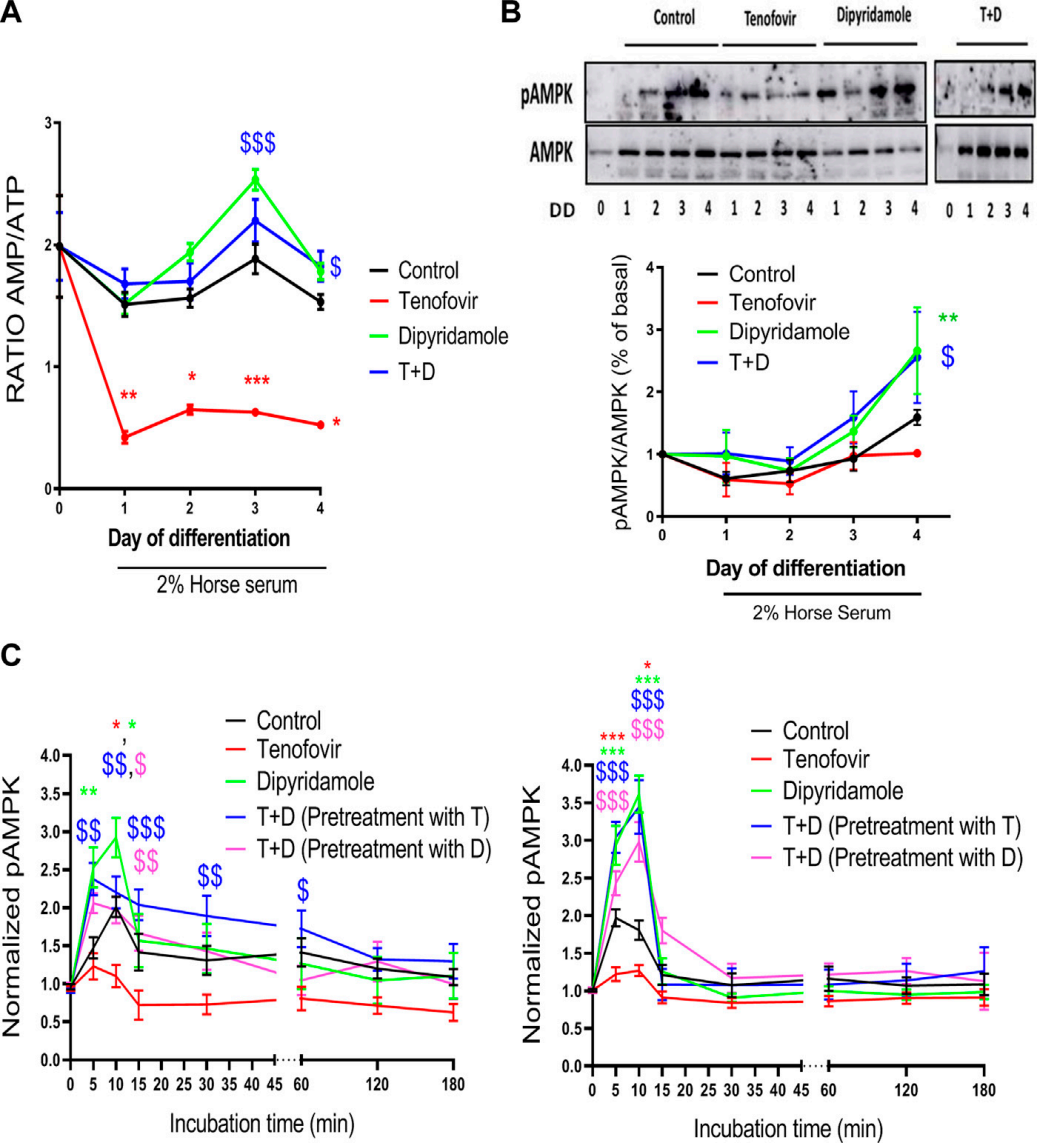
AMPK activation mediated by dipyridamole. **(A)** Ratio of intracellular AMP/ATP concentrations. Expression of phosphorylated **(B)** AMPK (pAMPK) and **(C)** pAMPK levels via ELISA in myoblasts (left) and myotubes (right). Data are presented as the mean ± SEM for *n* = 5–7 (Western blot and HPLC) or *n* = 9–12 (AMPK ELISA) per group and day. *: treatment group vs. control group; $: treatment group vs. tenofovir group. T + D: tenofovir + dipyridamole; DD: days of differentiation.

As AMPK activation depends on the intracellular AMP/ATP ratio, we established the AMP/ATP ratio according to the HPLC values. The AMP/ATP ratio remained stable over the 4 days of differentiation with 2% HS when compared to the basal ratio (*p = ns*), and it was significantly reduced with tenofovir (0.62 ± 0.03 tenofovir vs. 1.88 ± 0.12 control on day 3, *p < 0.001*, Figure 6A). Dipyridamole reverted the effect of tenofovir to control levels (2.19 ± 0.17 T + D vs. 0.62 ± 0.03 tenofovir, *p < 0.05*, Figure 6A), resulting in an AMP/ATP ratio similar to that of dipyridamole alone.

We next studied whether these changes in the AMP/ATP ratio had any effect on AMPK activation. Western blot analyses revealed no differences in pAMPK at Tyr172 over the 4 days of differentiation with 2% HS (Figure 6B), with no modulation by tenofovir (1.15 ± 0.05% tenofovir on day 4 vs. basal, *p = ns*, Figure 6B), while dipyridamole increased pAMPK on day 4 of differentiation when compared to the control (2.66 ± 0.7% dipyridamole vs. 1.29 ± 0.27% control on day 4 of differentiation, *p < 0.01*, Figure 6B). The combined treatment increased pAMPK on day 4 when compared to tenofovir (2.58 ± 0.73% T + D vs. 1.15 ± 0.05% tenofovir on day 4 of differentiation, *p < 0.05*, Figure 6B).

These results indicate that, in the long term, the decrease in the AMP/ATP ratio with tenofovir did not alter AMPK activation. To prove that AMPK activation occurred over short time points, AMPK activation assays, both at the myoblast and myotube stages, were performed. When we analyzed AMPK activation in myoblasts, AMPK activation increased 10 min after switching to 2% HS (1.95 ± 0.34 control vs. basal, *p < 0.05*, Figure 6C), with tenofovir keeping AMPK inactive (1.02 ± 0.19 tenofovir vs. 1.97 ± 0.17 control, *p = 0.05*, Figure 6C). Dipyridamole increased these levels after 5 and 10 min (2.89 ± 0.29 dipyridamole vs. 1.97 ± 0.17 control, *p < 0.05*, Figure 6C) and reverted the inactivation of AMPK induced by tenofovir (Figure 6C). Similar results were obtained when C2C12 cells were differentiated to myotubes for 4 days and then treated with tenofovir and dipyridamole for short amounts of time (Figure 6C).

### 3.6 Adenosine A2B receptor blockade reverses dipyridamole myogenesis modulation

**FIGURE 7:**
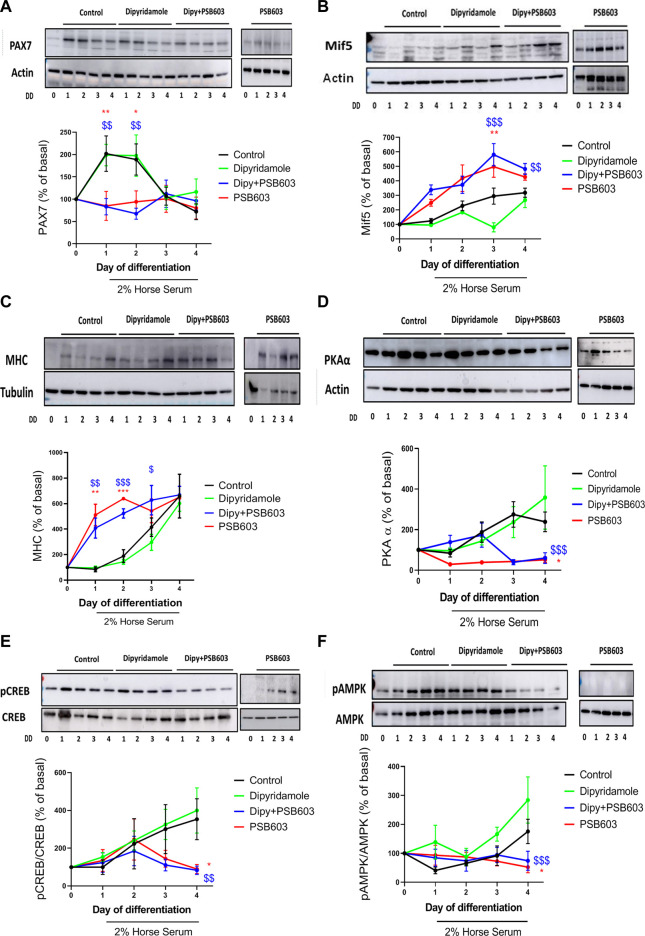
The effect of dipyridamole in myogenesis is dependent on the activation of the adenosine A2B receptor. Analysis of protein expression of **(A)** PAX7, **(B)** Mif5 and **(C)** MHC muscle proliferation/differentiation markers over 4 days of treatment with 2% HS + dipyridamole ± PSB-603; **(D)** protein expression of PKAα and **(E)** pCREB over 4 days of treatment with 2% HS + dipyridamole ± PSB-603; and **(F)** protein expression of pAMPK over 4 days of treatment with 2% HS + dipyridamole ± PSB-603. The results of the analysis are presented as the mean ± SEM *n* = 5 per group and day. *: treatment vs. control; $: dipyridamole vs. dipy + PSB-603; DD: days of differentiation.

The results shown in this manuscript indicate that dipyridamole exerted its effect by activating the A2B receptor. To prove this, we treated the cells with the selective A2B inhibitor PSB-603. PSB-603 completely reverted the increase in PAX7 induced by dipyridamole (83.2 ± 18.3% dipyridamole + PSB-603 vs. 198.7 ± 24% dipyridamole on day 1, *p < 0.005,* Figure 7A) in a similar manner to the inhibitor alone (Figure 7A). PSB-603 also reverted the decrease in Mif5 induced by dipyridamole (580 ± 76.4% dipyridamole + PSB-603 vs. 79.48 ± 31% dipyridamole on day 3, *p < 0.001*, Figure 7B), but it did not change MyoD and MyoG expression (Supplementary Figure S3). The PSB-603 pre-treatment led to the early expression of MHC (408 ± 78% dipyridamole + PSB-603 vs. 95 ± 13% dipyridamole on day 1, *p < 0.005*) in a similar manner to PSB-603 alone (Figure 7C).

The PSB-603 pre-treatment decreased PKAα expression when compared to dipyridamole (59 ± 26% dipyridamole + PSB-603 vs. 358 ± 156% dipyridamole on day 4, *p < 0.05*, Figure 7D), with a concomitant decrease in pCREB (79.6 ± 22.1% dipyridamole + PSB-603 vs. 399.6 ± 119.8% dipyridamole on day 4, *p < 0.005*, Figure 7E). PSB-603 also decreasedpAMPK expression (74.1 ± 33% dipyridamole + PSB-603 vs. 284 ± 79.8% dipyridamole on day 4, *p < 0.001*, Figure 7F). When cells were treated with a lower dose of PSB-603 (150 nM), similar results were observed, indicating a specific A2B receptor modulation by dipyridamole (data not shown).

## 4 Discussion

In the present manuscript, we have demonstrated that the modulation of the purinergic system alters muscular myogenesis. Our results suggest that the modulation of ATP output to the extracellular medium by tenofovir increases the intracellular ATP and decreases the extracellular adenosine. These produce a decrease of A2BR and inactivation of cAMP and AMPK, promoting premature muscle differentiation, reduction in PAX7 in the early stages of cell differentiation and an early appearance of the late muscle differentiation marker MHC, with no changes in the intermediate differentiation markers. On the other hand, dipyridamole which blocks the adenosine uptake by cells produces an increase of the available extracellular adenosine and intracellular AMP. This activates the adenosine A2B receptor, with increase of cAMP and AMPK levels which counteract the myogenesis alteration produced by tenofovir. Early muscle development has been shown to lead to the depletion of progenitor cells and the cessation of muscle growth (Schuster-Gossler et al., 2007). Maltzahn et al. showed that Pax7^−/−^ knockout mice showed early differentiation with loss of function of satellite cells during regenerative myogenesis (Maltzahn et al., 2013). Moreover, Liu et al. reported a reduction in the Pax7^+^ marker in the SAMP8 murine model of sarcopenia (Liu et al., 2020). Therefore, the regulation of the Pax7/MHC balance is fundamental for the correct myogenesis, and a reduction induced by tenofovir could favor the appearance of sarcopenic markers. Our data show that dipyridamole can reverse these early differentiation markers, acting as a counter-effector of tenofovir.

The nucleoside/nucleotide modulation by tenofovir and dipyridamole seems to be crucial for the changes observed in muscle myogenesis by these treatments. In general, an increase in both intra- and extracellular nucleotide levels has been observed with muscle differentiation. Increased nucleotide levels have previously been observed to favor differentiation in other models (Ryten et al., 2002; Orriss et al., 2006). Extracellular ATP levels have been shown to maintain muscle homeostasis during myogenesis (Martinello et al., 2011). Reduced levels of available adenosine have been linked to the development of muscle atrophy (Miller et al., 2019). This is in agreement with our data of early differentiation produced by tenofovir *in vitro,* as in the presence of this treatment, there is a decrease in adenosine and available extracellular ATP, concomitant with the accumulation of intracellular ATP in a very rapid and prolonged manner, and the decrease in the intracellular AMP levels over the final days of differentiation.

In previous studies, it was shown that the release of ATP was necessary for the formation of the myotube (Martinello et al., 2011). However, the intracellular ATP levels in myoblasts and myotubes were not studied. We demonstrated that pharmacological accumulation of ATP, mediated by tenofovir, leads to the expression of late differentiation markers in the early stages of differentiation. On the other hand, dipyridamole reverses nucleotide levels and increases the levels of available extracellular adenosine preventing myogenesis alterations. When cells were treated with exogenous adenosine or ATP, we observed that 100 µM ATP mimics tenofovir (Supplementary Figure S4) meanwhile exogenous adenosine (7.5 µM) administration did not modulate the myogenesis and did not replicate AMPK nor PKAα stimulation as dipyridamole neither alone or in the presence of EHNA 1 µM(Supplementary Figure S4, S5). This effect might be explained due to the inespecificity of adenosine receptor activation (Verani, 1991). Adenosine has a higher affinity for the A1 adenosine receptor compared to the other adenosine receptors (Stockwell et al., 2017). The inactivation of adenylate cyclase and thus the reduction of cAMP formation by the adenosine A1 receptor explains why PKAα expression is not increased with the use of exogenous adenosine (Hershberger et al., 1991). On the other hand, the reduced formation of cAMP would reduce the formation of intracellular 5′-AMP and AMPK would be inactivated by the reduction of the AMP/ATP ratio (Salminen and Kaarniranta, 2012; Ke et al., 2018). On the other hand, the use of exogenous ATP leads to an increase in MHC expression 24 h after treatment, which replicates the premature differentiation observed by tenofovir. The use of exogenous ATP had already been shown to potentiate osteoblastic differentiation (Stovall et al., 2020). In addition, ATP leads to an inactivation of AMPK. The use of exogenous ATP is possible that produces an increment of ATP/AMP ratio which led to inactivation of AMPK (Ke et al., 2018). Also, ATP decreased the PKAα expression. AMPK and cAMP interplay in many signaling pathways. Therefore, the decreased levels of AMPK is possible that modulates cAMP and produces a decrease in the PKAα expression (Zhang et al., 2017).

In human skeletal muscle, both the A2A and A2B adenosine receptors are localized, but the A1 receptor is not (Lynge and Hellsten, 2000). In C2C12 cells, we have verified that both the gene and protein levels of the four adenosine receptors can be detected. A1 and A2A play an antagonistic role in myogenesis. A1 increases with cell differentiation, while A2A is only detectable when the muscle is in a proliferative state. This correlates with previous studies that detected a greater amount of A2A than A1 in C2C12cells (Gnad et al., 2020) and supports the proliferative role of A2A and the differentiation role of A1. Furthermore, in cancer, it is known that the fundamental role of the A2A receptor is to promote tumor proliferation by inhibiting the immune response (Sun et al., 2022). However, we did not see any effect with tenofovir or dipyridamole, suggesting that the modulatory effect of dipyridamole and the available adenosine is mediated by another adenosine receptor. It has been recently demonstrated that the adenosine A2B receptor is the most expressed of all receptors in human skeletal muscle and is related to the maintenance of satellite cells with age, the delay of muscle senescence and the improvement of strength (Gnad et al., 2020). In our case, we observed an increase in the adenosine A2B receptor levels with muscle differentiation, which was lost with tenofovir. Dipyridamole is capable of accentuating the increase in adenosine A2B expression and recovering the levels lost by tenofovir. This suggests that the increase in available extracellular adenosine by dipyridamole is captured by the major receptor in the line, and this is capable of activating secondary effectors. During differentiation, we also found an increase in the expression of the adenosine receptor A3 with myogenesis, which was lost in the dipyridamole treatment. This increase could be due to the level of adenosine during differentiation (<15 nM). Previous studies showed that A3 stimulation with similar levels of adenosine had an anti-proliferative role. Therefore, it is possible that the increase in the A3 receptor during differentiation counteracts the proliferative state in C2C12 cells (Mazziotta et al., 2022). However, this receptor is minor compared to the rest, and it is suggested that it therefore has lower functionality in muscle (Gnad et al., 2020).

The modulation of intracellular AMP and ATP levels by tenofovir and dipyridamole could serve as a key to the transition from catabolism to energy anabolism through the activation of AMPK. We verified that AMPK phosphorylation progresses with the days of differentiation. For its part, tenofovir leads to a loss in the AMP/ATP ratio, which we observed to be capable of inhibiting AMPK in both myoblasts and myotubes. Dipyridamole recovers AMP/ATP levels, maintaining AMPK activation in both models. Emerging studies indicate that the responsiveness of AMPK signaling clearly declines with aging (Salminen and Kaarniranta, 2012). All of these findings suggest a catabolic role for tenofovir and an anabolic role for dipyridamole (Herzig and Shaw, 2018).

Adenosine receptors play an antagonistic role in the modulation of cAMP (Singh et al., 2018). The activation of the A1/A3 receptors inhibits cAMP, and the activation of the A2A and A2B receptors promotes cAMP (Singh et al., 2018). Response-element binding-protein (CREB) phosphorylation due to increased cAMP and, subsequently, PKAactivation has been related to increased proliferation in muscle myogenesis (Chen et al., 2005), hypertrophy/cell migration (Berdeaux and Stewart, 2012) and regeneration of damaged muscle (Stewart et al., 2011). Therefore, elucidating the role of tenofovir and dipyridamole in the activation of cAMP and its effectors is essential to understand their modulator role in myogenesis. *In vitro*, we observed that tenofovir did not modulate cAMP levels, which is explained by the reduction in available extracellular adenosine. Dipyridamole reversed this effect by producing cAMP activation in myoblastsandmyotubes. cAMP has recently been proposed as a molecule that improves motor activity as well as delaying the effects of aging (Wang et al., 2015). It has been observed by Wang et al. that daily treatment with cAMP in 24-month-old mice led to a delay in the appearance of the age-related phenotype as well as an improvement in motor activity (Wang et al., 2015). On the other hand, inhibition of adenosine A2B receptor expression led to a decrease in cAMP not seen with adenosine A2A receptor inhibition *in vitro* (Gnad et al., 2020). The use of inhibitors of phosphodiesterase-4 (PDE 4), the major cAMP-modifying PDE found in skeletal muscle, reduces the loss of muscle mass and force resulting from denervation in rat and mouse models (Hinkle et al., 2005). All of these findings suggest that cAMP prevents muscle aging, and dipyridamole may contribute to muscle maintenance in aging.

Activation of cAMP with dipyridamole leads to increased PKAα expression and CREB phosphorylation. The PKA α-subunit is critical for structural conformation and activity homeostasis of cAMP (Haushalter et al., 2018). An increase of cAMP activity and PKA regulatory α-subunit is observed with dipyridamole compared with tenofovir. Zhan et al. explained that a decrease in PKA α-subunit activation causes an aberrant cAMP signaling, due to an ineffective cAMP compartmentation (Zhang et al., 2020). Furthermore, PKA α-subunit is known that primes the PKA catalytic activity (Haushalter et al., 2018). Therefore, we propose that tenofovir produce an aberrant activity of cAMP due to a decrease of PKA α-subunit, accompanied with decrease expression of CREB and increase of EPAC2. The CREB transcription factor, a final effector of cAMP signaling, has been related to increased myoblast proliferation as well as the expression of early myogenic transcription factors in cultured primary myocytes (Stewart et al., 2011). This correlates CREB activation with muscle regeneration after damage (Stewart et al., 2011). Therefore, we can predict that the increase in pCREB via cAMP by dipyridamole could improve the proliferative capacity of myoblast cells during muscle regeneration.

This study presents some limitations. Herein we demonstrated that dipyridamole exerts an activation of the adenosine A2B receptor, however, study of the effect of dipyridamole on other pathways would be of interest. Dipyridamole is not understood as a modulator of myogenesis *per se*, but it counteracts alterations in muscle myogenesis, such as premature differentiation induced by tenofovir-mediated ATP transport blockade. Therefore, is necessary test if these myogenesis modulations occur in an*in vivo*model of sarcopenia. Moreover, there are differences in the metabolism of human muscle cells and the C2C12 line. Because of this heterogeneity it would be of interest test the effect of dipyridamole in human muscle cells (Abdelmoez et al., 2020). Finally, extracellular AMP levels were not ted at extracellular levels by HPLC. Likewise, no modulation of ADP levels was observed. We have demostrated that myogenesis induce an increase of protein expression in both CD39 and CD73 but these enzymes are not modulated neither by dipyridamole, nor tenofovir. This correlates with previous data where we previously demonstrated that we need a high concentration of tenofovir to inhibit ATP dephosphorylation and we concluded that tenofovir did not inhibit the conversion of adenine nucleotides to adenosine (Feig et al., 2017).

We conclude that adenosine and ATP act as mediators in muscle myogenesis. Modulation of the purinergic system with compounds that increase extracellular adenosine levels, such as dipyridamole, produces an activation of the A2B adenosine receptor and an increase in cAMP and AMPK signaling. These pathways, known for their anti-aging effects in muscle, support the role of dipyridamole as a good therapeutic approach in sarcopenia.

## Data Availability

The raw data supporting the conclusion of this article will be made available by the authors, without undue reservation.

## Supplementary material

SUPPLEMENTARY FIGURE S1 Effect of tenofovir or dipyridamole on C2C12 cells. Cell proliferation capability was assessed using an Alamar Blue dose–response assay after 24 and 48 h of treatment of the C2C12 cell line with tenofovir or dipyridamole. Data are presented as the mean ± SEM for *N* = 8 per group.

SUPPLEMENTARY FIGURE S2 Real-time RT-PCR analysis over the 4 days of differentiation in the presence of tenofovir and dipyridamole. mRNA expression of the A1, A2A, A2B, and A3 adenosine receptors, P2X7 and Panx-1 is shown. Data are the mean ± SEM (*n* = 5 per group and day). *: treatment vs. basal; $: treatment vs. control; #: dipyridamole and T+D vs. tenofovir; DD: days of differentiation.

SUPPLEMENTARY FIGURE S3 Protein expression of early muscle differentiation markers. Western blot analysis of MyoD and MyoG in C2C12 + 2% HS ± dipyridamole and PSB-603 for 4 days. Dataare presented as the mean±SEM (*N* = 5 per group and day).*:treatment vs. control; $: dipyridamole vs. dipy+PSB-603; DD: days of differentiation.

SUPPLEMENTARY FIGURE S4 Exogenous administration of adenosine and ATP to C2C12 modulates myogenesis and AMPK/PKAα activation. Western Blot analysis for PAX7, MHC, AMPK and PKAα at basal, 24 h and 4 days of differentiation. One representative of *n* = 5 per group is displayed. Data are the mean ± SEM. *: treatment vs. basal; #: between treatments. h: hours of differentiation; D: day of differentiation.

SUPPLEMENTARY FIGURE S5 Exogenous adenosine + EHNA do not modulate the myogenesis of C2C12 cell line. Western Blot analysis for PAX7, MHC, AMPK and PKAα at basal, 24 h and 4 days of differentiation. One representative of *n* = 5 per group is displayed. Data are the mean ± SEM. *: treatment vs. basal; h: hours of differentiation; D: day of differentiation.
