# Supplementary figures and images for: Dipyridamole activates adenosine A2B receptor and AMPK/cAMP signaling and promotes myogenic differentiation of myoblastic C2C12 cells
Source: Front Pharmacol. 2023 Sep 12;14:1247664. doi: 10.3389/fphar.2023.1247664 (PMC10522837; doi:10.3389/fphar.2023.1247664)

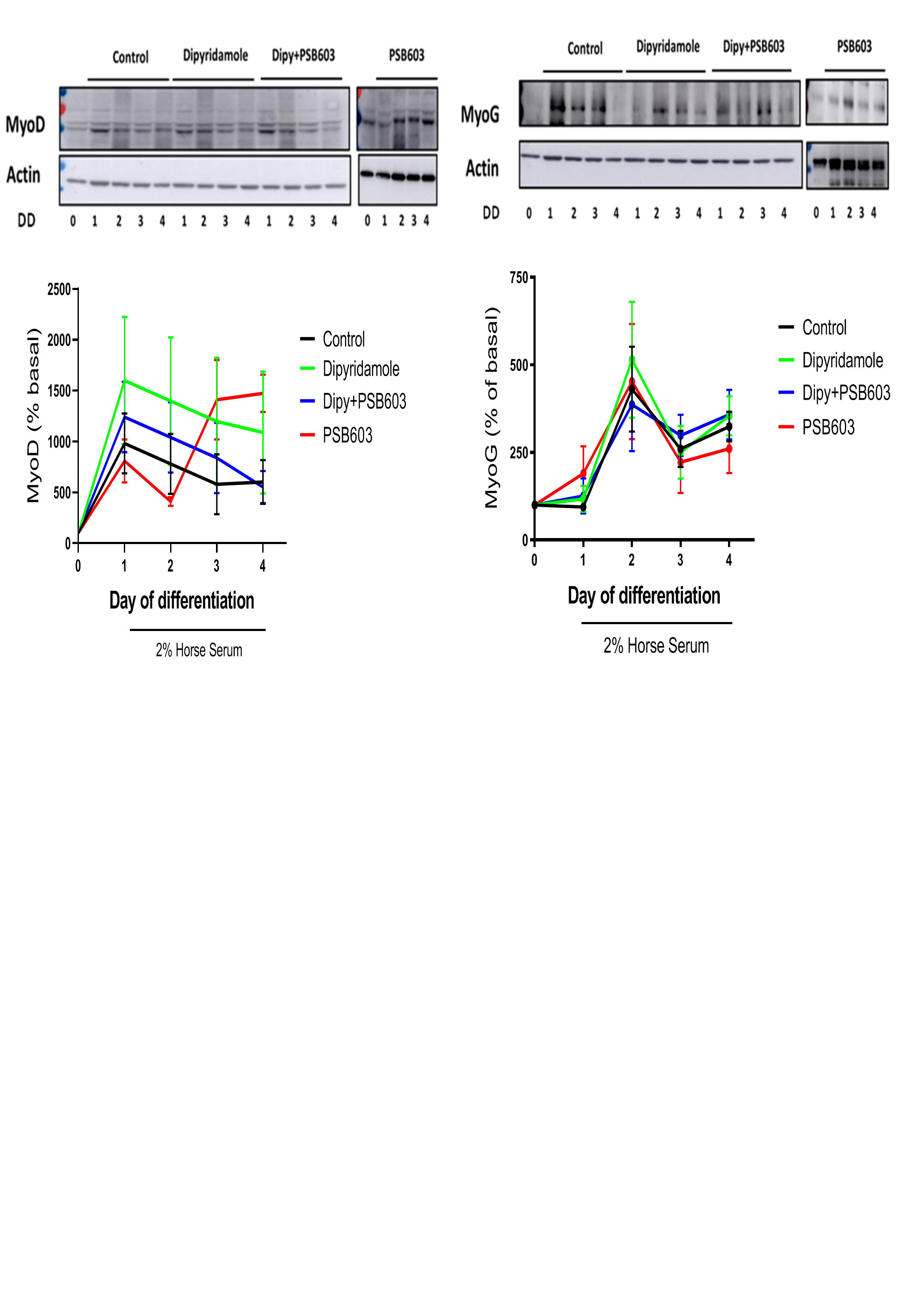

Supplement: Supplementary file 1 [file Image3.tif]

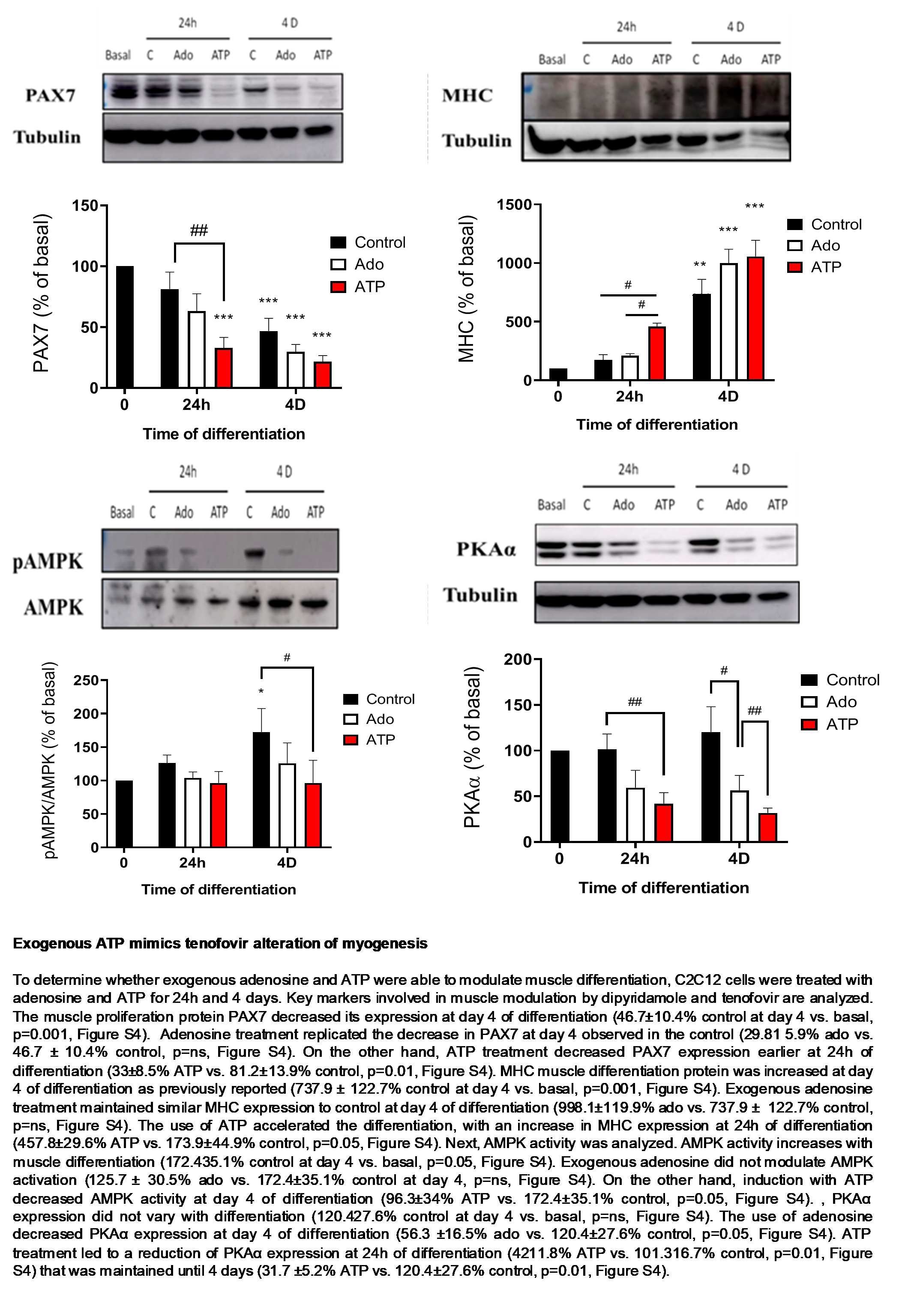

Supplement: Supplementary file 2 [file Image4.tif]

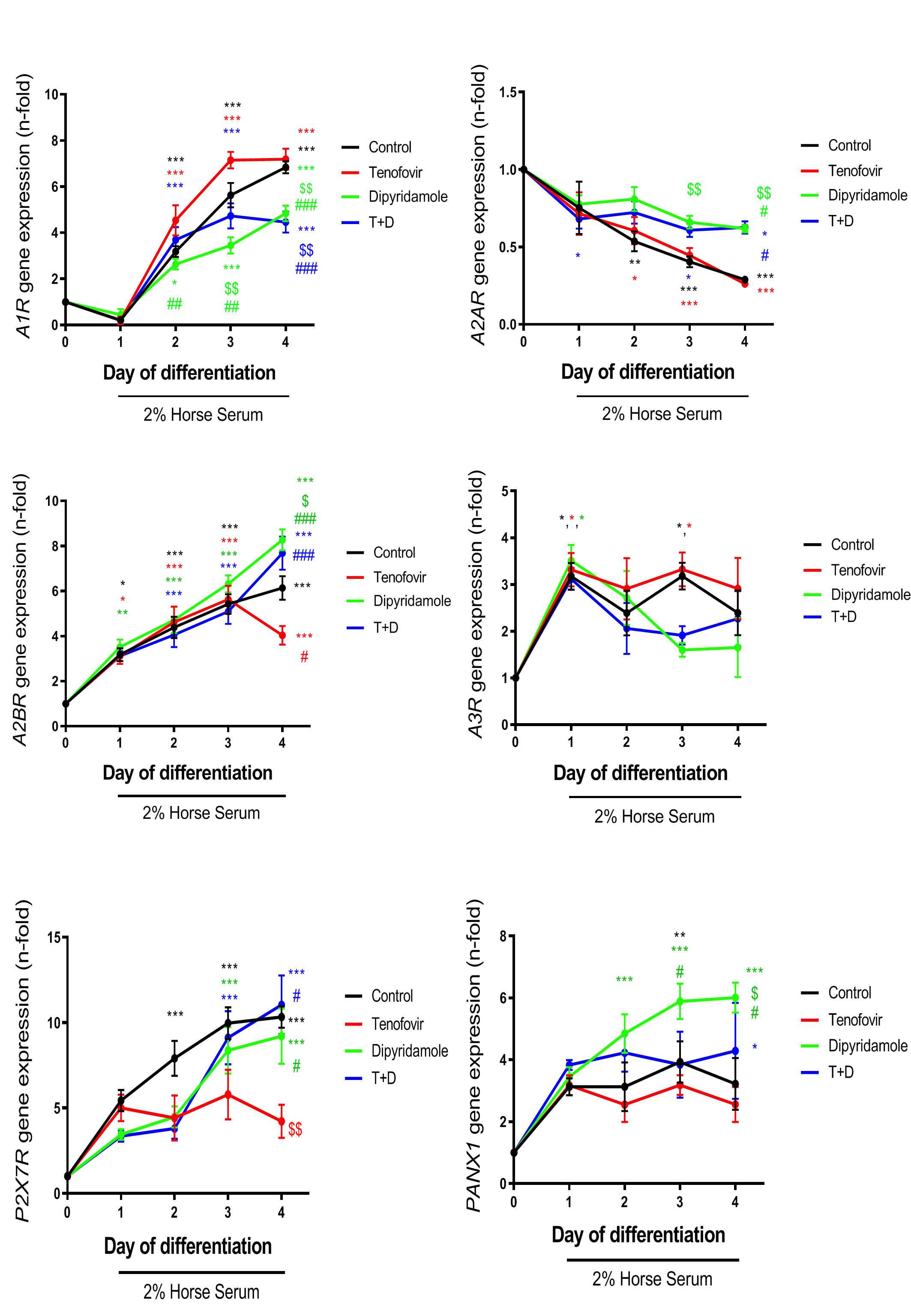

Supplement: Supplementary file 3 [file Image2.tif]

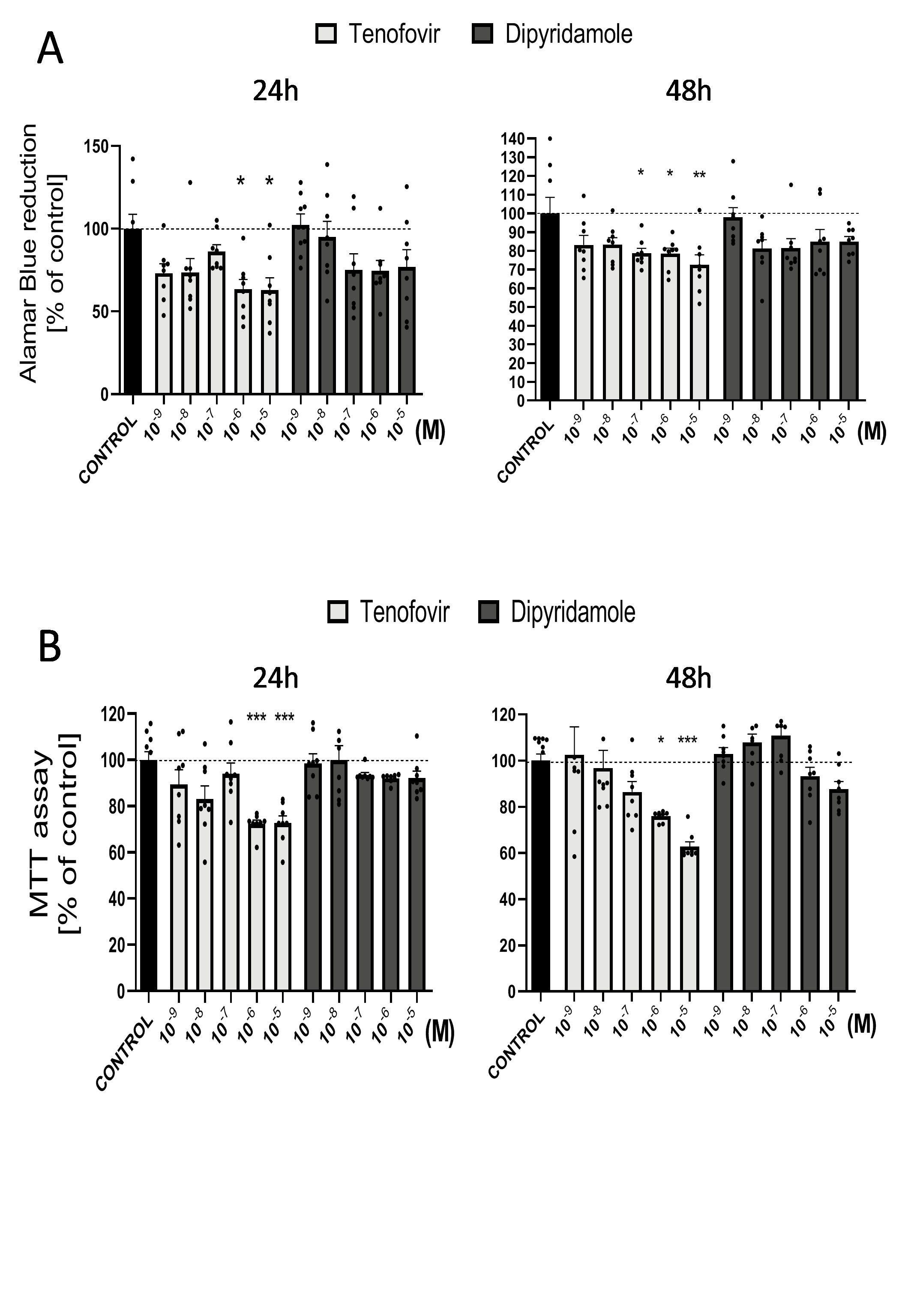

Supplement: Supplementary file 4 [file Image1.tif]

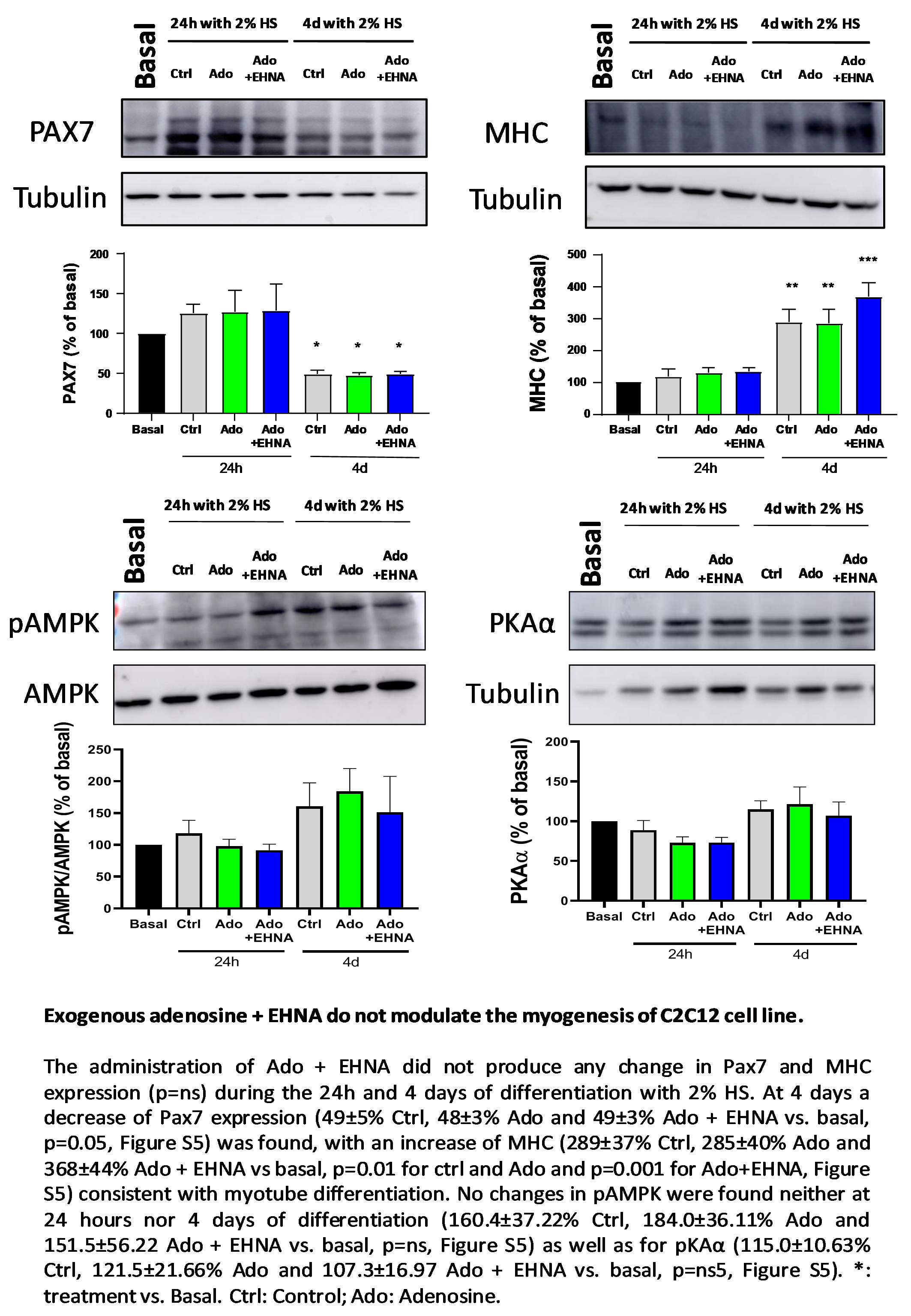

Supplement: Supplementary file 5 [file Image5.tif]
